# Supplementary material for: Learning curve and surgical outcome of robotic assisted colorectal surgery with ERAS program
Source: Sci Rep. 2022 Nov 29;12:20566. doi: 10.1038/s41598-022-24665-w (PMC9709162; doi:10.1038/s41598-022-24665-w)
Supplement: Supplementary file 2 — Supplementary Information 2. [file 41598_2022_24665_MOESM2_ESM.zip › RAL Raw data-Ñ[▒K/117/HS_ñw╝╨░O▒Kñσ.pdf]

正式摘要(電子簽章：00000227095338)		
W83 / W83 ☐ 英文標題(注意:無英文姓名-請補病人基本檔!)	☐【讀取】憑證	

臺中榮民總醫院

出院病歷摘要

轉入號 : 轉出號 :

[redacted]

**(8)**流水編號	
**(9)**入院日期	110/10/08 (2021) 直腸外科 W83 - 071
**(10)**轉科日期	
**(11)**出院日期	110/10/13 (2021) 住院天數計 5 日

診斷

- 入院 :Sigmoid colon cacncer
- 出院 :Sigmoid colon cancer s/p robotic anterior resection on 2021/10/9

主訴

- bloody stool for several days

病史

- This 45 years old female patient didn't have special past history. She complained lower abdominal dull pain and bloody stool for several days. She came to 光田H for help. Colonoscopy showed tumor at Sigmoid colon that malignancy was proved in biopsy report. Thus, she came to our hospital. After complete survey, she was admitted for surgical intervention.

體檢發現

-
  - General appearance :
    - easy looking , conscious clear , without cardiopulmonary distress
    - Vital signs自動帶入date 體溫(BT) 脈搏(P) 呼吸(R) 血壓(BP) 身高(BH) 體重(BW) BMI
      20211008 35.9℃ 78次/分 18次/分 133/75mmHg 158.1Cm 60.3Kg 24.1
  - HEENT :
    - Eyes: no anemic conjunctiva, no icteric sclera, normal EOM
    - ENT: no otorrhea, no rhinorrhea, no erythematous mucosa, no injected tonsil, no deformity
  - Neck: supple, freely movable, trachea midline,no deformity, no engorged jugular vein , no palpable thyroid or lymph node enlargement
  - Chest:
    - Inspection: Grossly normal, symmetric and fully expansion, no accessory muscle used, no operation scar, no spider angiomata, no gynecomastia
    - Palpation: no palpable mass or nodule, no subcutaneous emphysema, no tenderness
    - Ausculation: clear breathing sound
  - Heart:
    - Percussion: normal heart size
    - Ausculation: regular heart beat, normal S1, S2, no gallop, no murmur
  - Abdomen:
    - Inspection: in
      - flat contour, symetrically, no scar, no stria, no dilated veins, no rashes
    - Ausculation: normoactive bowel sound, no abdominal bruits
    - palpation: soft, no muscle guarding or rigidity, no local tenderness, no rebounding pain, no palpable mass, no palpable pulsation
    - Percussion: no shifting dulllness, no hepatomegaly, no splenomagaly
  -

Peripheral pulsation :	CA	BA	RA	FA	PA	PTA	DPA

-

Right side :	++	++	++	++	++	++	++/+++
Left side :	++	++	++	++	++	++	++/+++

- Genito-anus: no tenderness or palpable mass lesion or rectal shelf, normal external genitalia, normal anal tone, brown stool

CRS CONDITION

- CEA: 1.95 ng/ml
-
    - Lung

手術日期及方法

- 手術日期及方法20211009 1. Robotic (XI) assisted anterior resection
  2. Intraoperative colonoscopy : (-).
  3. High ligation (+) : At IMA root. Left colic artery and IMV were also ligated via Endoclips.
  4. Take down from the splenic flexure : (-)
  5. Robotic endoGIA (45-3.5) X1 + EEA (29mm) were used
  6. Proximal cut end: 10 cm, distal cut end: 2 cm
  7. No drainage tube was inserted

住院治療經過

- After admission, operation with robotic anterior resection was done on 10/9. We let her try water and then diet under ERAS protocol. Under the stable condition, she was discharged on 10/13.

合併症

- nil

一般檢查

- 累積報告 :

DATE	TIME	ALB	TP	BIL.T	BIL.D	ALKP	AST	ALT	LDH	AMY	LIPASE	NH3	Ferritin	UA
1101009	1450	3.8		0.5				10						
1101013	0852	3.1		0.41	0.11		15	6						

DATE	TIME	WBC	RBC	HGB	HCT	MCV	PLT	BLST	NEUT	BAND	SEG	LYM	MON	EOS	BAS
1101009	1449	10030		12.5	38.6		213		92.2			6.3	1.4	0.0	0.1
1101013	0856	4830	4.26	12.0	37.3	87.6	209		57.3			28.2	6.8	7.5	0.2

DATE	TIME	NA	K	CL	BUN	CREAT	eGFR	CA	Mg	P	CRP	ESR	PCT	D-Dimer	LACTATE
1101009	1450	144	4.1	111	11	0.69	97.79	8.7							
1101013	0852	140	3.8		5	0.61	112.73	8.0							

特殊檢查

病理報告

其它

出院時情況

- 3.改門診治療

出院指示

-

藥名	劑量	劑量單位	用法	頻次	數量	數量單位	現況	天數	單位
(口)Takepron OD tab 30mg	1	TAB	PO	QDAC	7	TAB	TH	7	天
Ultracet tab	1	TAB	PO	TIDPRN	21	TAB	TH	7	天
Mag. Oxide tab	2	TAB	PO	TID	84	TAB	TH	14	天

出院計劃

主治醫師 2062F 蔣鋒帆 / 住院醫師 2331F 陳凱祥

正式摘要(電子簽章：000000227095338)

W83 / W83

doctorRank:B
